# Molecular Cytogenetic Characterization of two *Triticum–Secale–Thinopyrum* Trigeneric Hybrids Exhibiting Superior Resistance to Fusarium Head Blight, Leaf Rust, and Stem Rust Race Ug99

**DOI:** 10.3389/fpls.2017.00797

**Published:** 2017-05-15

**Authors:** Yi Dai, Yamei Duan, Huiping Liu, Dawn Chi, Wenguang Cao, Allen Xue, Yong Gao, George Fedak, Jianmin Chen

**Affiliations:** ^1^College of Bioscience and Biotechnology, Co-Innovation Center for Modern Production Technology of Grain Crops, Yangzhou UniversityYangzhou, China; ^2^Ottawa Research and Development Centre, Agriculture and Agri-Food Canada, OttawaON, Canada; ^3^Jiangsu Key Laboratories of Crop Genetics and Physiology, Plant Functional Genomics of the Ministry of Education, Yangzhou UniversityYangzhou, China

**Keywords:** Fusarium head blight, leaf rust, Ug99, trigeneric hybrid, triticale, genomic *in situ* hybridization

## Abstract

Fusarium head blight (FHB), leaf rust, and stem rust are the most destructive fungal diseases in current world wheat production. The diploid wheatgrass, *Thinopyrum elongatum* (Host) Dewey (2*n* = 2*x* = 14, EE) is an excellent source of disease resistance genes. Two new *Triticum–Secale–Thinopyrum* trigeneric hybrids were derived from a cross between a hexaploid triticale (X *Triticosecale* Wittmack, 2*n* = 6*x* = 42, AABBRR) and a hexaploid *Triticum trititrigia* (2*n* = 6*x* = 42, AABBEE), were produced and analyzed using genomic *in situ* hybridization and molecular markers. The results indicated that line RE21 contained 14 A-chromosomes, 14 B-chromosomes, three pairs of R-chromosomes (4R, 6R, and 7R), and four pairs of E-chromosomes (1E, 2E, 3E, and 5E) for a total chromosome number of 2*n* = 42. Line RE62 contained 14 A-chromosomes, 14 B-chromosomes, six pairs of R-chromosomes, and one pair of translocation chromosomes between chromosome 5R and 5E, for a total chromosome number of 2*n* = 42. At the seedling and adult growth stages under greenhouse conditions, line RE21 showed high levels of resistance to FHB, leaf rust, and stem rust race Ug99, and line RE62 was highly resistant to leaf rust and stem rust race Ug99. These two lines (RE21 and RE62) display superior disease resistance characteristics and have the potential to be utilized as valuable germplasm sources for future wheat improvement.

## Introduction

Fusarium head blight (FHB), caused by *Fusarium graminearum*, *F. culmorum*, and other *Fusarium* species, is one of the most destructive fungal world diseases in wheat production. FHB outbreaks reduce grain yield and quality in both common wheat (*Triticum aestivum*) and durum wheat (*T. durum*) and can contaminate the grain with mycotoxins (Arseniuk et al., 1993; Kalih et al., 2014; Zhu et al., 2015). Currently, the most effective and widely used quantitative trait locus (QTL) for FHB resistance breeding is located on chromosome 3BS of the Chinese wheat varieties Sumai 3 and Wangshuibai. Recent investigations of several other wild relatives of wheat, such as diploid wheatgrass *Leymus racemosus*, *Th. intermedium*, *Th. elongatum*, and tetraploid wheatgrass *Th. junceiforme*, have been shown to be highly resistant to FHB (Chen et al., 2005; Becher et al., 2013; Kalih et al., 2014). Additionally, some wheat-wild species, including accessions of the St genome of *Th. intermedium* and the E genome of *Th. elongatum*, have been shown highly resistant to FHB, making these species the most successful examples for introgression of elite genes from wild relatives for wheat improvement (Jauhar et al., 2009; Scoles et al., 2009; Zeng et al., 2013).

Leaf rust is caused by the fungus *Puccinia triticinia* Eriks. (*Pt*) and is the most common and widespread disease in wheat (Anikster et al., 1997). Losses from leaf rust infection are usually less than those from stem rust and stripe rust, but leaf rust occurs more frequently over a larger area, resulting in greater annual losses (McCallum and Seto-Goh, 2005). Various strategies have been developed to control leaf rust, the most economical and effective of which has been to use new resistance resources of cultivated germplasm (Chu et al., 2009). Stem rust is caused by *P. graminis* f. sp. *tritici* (*Pgt*) and is an economically impactful disease of wheat that can be controlled by using effective stem rust resistance (*Sr*) genes. However, a new stem rust race, commonly known as Ug99 and formally designated as TTKSK, was detected in Uganda in 1999 that is virulent to plants carrying *Sr31* and many other *Sr* genes (Jin et al., 2007, 2008). Singh et al. (2011) heeded Dr. Norman Borlaug’s warning in 2005 that stem rust race Ug99 could spread throughout the world wheat production areas, leading to worldwide food shortages, because approximately 85–95% of globally distributed common wheat cultivars are susceptible to stem rust race Ug99. For economic and environmentally sustainable disease control, Ug99-resistant wheat varieties should be developed from cultivated germplasm or wild related species. Zeng et al. (2013) obtained translocation lines from the E^e^ and St genomes of *Th. intermedium*, and these lines have shown good resistance to leaf rust and stem rust race Ug99.

Distant hybridization is one way to trigger reorganization of different genomes in hybrids (Fu et al., 2013). Wild relatives of common wheat, which have superior agronomic traits and resistance to biotic and abiotic stresses, are often used as desirable gene donors in wheat breeding programs (Dong et al., 1992; Mujeeb-Kazi et al., 2013). To date, many genes from wild species conferring superior traits have been transferred into cultivated wheat by developing newly synthesized allopolyploids (Qi et al., 2016). For example, breeders developed a man-made crop, triticale (X *Triticosecale* Wittmack), by crossing wheat (*T. turgidum* or *T. aestivum*) and rye. It combines the biotic and abiotic stress tolerances of rye and grain production ability of wheat, making it more suitable for production in agriculturally marginal areas (Mergoum and Macpherson, 2004). Although the rye genome carries many disease resistance genes, the global supply of triticale has inherent problems, including partial floret sterility, shriveled seed, production concerns, requirement for a long growing season and susceptibility to FHB as common wheat (Blum, 2014). Hence, there is a need to improve the genetic diversity and enhance the resistance to FHB of triticale by transferring desirable genes from additional wild relatives of wheat. Trigeneric hybrids could help establish evolutionary relationships among different genomes present in the same genetic background and could also be used as bridges to transfer different genes from wild species into cultivated wheat (Kang et al., 2012). Many trigeneric hybrids have been produced by crossing intergeneric hybrids or amphiploids with another species, such as *Triticum*–*Secale*–*Agropyron* (Gupta and Fedak, 1986), *Triticum*–*Secale*–*Aegilops* (Orellana et al., 1989), *Triticum*–*Secale*–*Hordeum* (Fernandez-Escobar and Martin, 1989), *Triticum*–*Secale*–*Thinopyrum* (Yu et al., 1993), and *Triticum*–*Secale*–*Leymus* (Li et al., 2006). These trigeneric hybrids have been used as bridges to transfer useful genes or characters from wild species into wheat (Jiang et al., 1993).

The diploid wheatgrass *Th. elongatum* (Host) D.R. Dewey [=*Lophopyrum elongatum* (Host) Á. Löve; =*Elytrigia elongatum* (Host) Nevski; =*Agropyron elongatum* (Host) Beauv.] (*2n* = 2*x* = 14, EE) is a significant wild relative of wheat that has many desirable genes for wheat improvement. Its high cross-compatibility with wheat and its superior value-added trait characters, especially FHB resistance has attracted the attention of wheat geneticists and breeders (Shukle et al., 1987; Sharma et al., 1989; Yang and Ren, 2001; Shen et al., 2004; Linc et al., 2012; Chen et al., 2013). Shen et al. (2004) reported that three substitution lines 7E(7A), 7E(7B), and 7E(7D) showed significant resistance to FHB, indicating that the 7E chromosome of *Th. elongatum* carries FHB resistance genes. Jauhar et al. (2009) obtained a stable durum 1E disomic addition line which showed significant resistance to FHB in greenhouse conditions, so it is indicated that the 1E chromosome of *Th. elongatum* may also carry FHB resistance genes. In this study, we obtained two stable lines RE21 and RE62, which were derived from the cross between hexaploid triticale (AABBRR) and hexaploid *T. trititrigia* (AABBEE). And these two lines displayed great resistance to leaf rust and stem rust race Ug99; line RE21 also had FHB resistance. The chromosome composition of these two lines was distinguished using chromosome-specific molecular markers and genomic *in situ* hybridization (GISH). These new lines will be useful for triticale breeding to transfer multi-alien genes into hexaploid common wheat, extend genetic resources for wheat breeding, enrich the genetic base of wheat, and improve wheat yield and quality.

## Materials and Methods

### Plant Materials and Molecular Markers

The genetic stocks used included common wheat Chinese Spring, Thatcher, Morocco, Hoffman, Chinese Spring-*Th. elongatum* addition lines and Chinese Spring-Imperial addition lines, An 8455, Sumai 3, Roblin, durum wheat (cv. Langdon), rye (Imperial), *Th. elongatum*, hexaploid triticale T182 (*2n* = 6*x* = 42, AABBRR), and hexaploid *T. trititrigia* 8801 (*2n* = 6x = 42, AABBEE). This plant material is stored at Ottawa Research and Development Center, Canada. The chromosome-specific molecular markers for rye and *Th. elongatum* were designed by Dai (Supplementary Table S1) and Chen et al. (2013), respectively, based on SLAF-seq technology (Supplementary Table S1).

Lines RE21 and RE62 were derived from a cross between 8801 × T182 at Ottawa Research and Development Center, Canada. F_1_ plants were obtained by embryo rescue and grown in 6-inch pots in the greenhouse under controlled conditions of 15°C for 8 h in the dark followed by 20°C for 16 h of light. The F_2_ to F_5_ lines were selected based on relative fertility and the severity of disease phenotype in the field. Seeds collected from selected F_6_ to F_8_ lines were used for molecular marker analysis and cytological studies. Adult F_6_ to F_8_ lines were evaluated for agronomic performance and disease resistance in the field and greenhouse conditions.

### Genomic DNA Extraction and Polymerase Chain Reaction (PCR) Analysis

Genomic DNA was extracted from 2 g of fresh leaves using the SDS–phenol–chloroform method (Sharp et al., 1989; Devos et al., 1992) and purified to eliminate RNA. DNA quality and concentration were measured by ethidium bromide visualization after 0.8% agarose gel electrophoresis. DNA was diluted to a final concentration of 100 ng/μL and stored at -20°C until use.

PCR amplification was carried out in 25 μL reactions containing 1 μL genomic DNA (100 ng/μL), 2.5 μL PCR buffer (10×), 2 μL dNTPs (2.5 mM total), 1 μL each of primer 1 (10 mM) and primer 2 (10 mM), 0.2 μL Taq DNA polymerase (5 U/μL), and 18.3 μL double-distilled water. The PCR cycling program was as follows: 94°C for 5 min, followed by 35 cycles at 94°C for 45 s, appropriate annealing temperature (based on different primers) for 45 s, 72°C for 40 s, with final extension at 72°C for 10 min.

### Molecular Cytogenetic Analysis

Chromosomes were prepared from mitotic metaphase following the protocol described by Chen et al. (2005). GISH was performed using techniques described by Zhao et al. (2013). Total genomic DNA of Imperial rye and *Th. elongatum* were labeled with DIG-Nick Translation Mix and Biotin-Nick Translation Mix (Roche, Germany), respectively, for use as probes for multicolor GISH. Hybridization signals were observed using a Zeiss Axioplan 2 fluorescence microscope, and images of signal patterns were acquired with a charge-couple device camera (Zeiss, Germany).

### Evaluation of Disease Resistance

Trigeneric hybrid lines RE21 and RE62 were screened for FHB resistance in the field and greenhouse. At anthesis, one floret in the middle of each spike was injected with 10 μL of an inoculum (50,000 spores/mL) mixture of three *F. graminearum* isolates: DAOM178148, DAOM232369, and DAOM212678 (Canadian Collection of Fungal Cultures at the Ottawa Research and Development Centre, Ottawa, ON, Canada), which were chosen as they are known to be aggressive (Xue et al., 2004), and at least three spikes from each plant was injected. Following inoculation, the plants were misted for 48 h (15 s of misting every 15 min) and maintained at 25°C for FHB development. After 21 days, all infected spikelets per inoculated spike were counted. Wheat cultivars An 8455 and Roblin served as the susceptible controls in both the field and greenhouse, respectively, and Sumai 3 served as the resistant control in both the field and greenhouse. Data were analyzed for statistical significance using ANOVA tests (Software package 21.0, SPSS).

Lines RE21 and RE62 were tested at the seedling stage for resistance against the five *P. triticinia* (*Pt*) races MBDS, TJBJ, MGBJ, MBRJ, and TDBG, which were obtained from the Canadian Collection of Fungal Cultures at the Ottawa Research and Development Centre, Ottawa, ON, Canada (Kolmer, 2001); and one *P. graminis* f. sp. *tritici* (*Pgt*) race, TTKSK (Ug99). The resistance to steam rust race Ug99 was tested in an level-3 containment facility (lab) at Agriculture and Agri-Food Canada, Morden Research and Development Centre, Morden, MB, Canada, and the method was followed by Fetch et al. (2009). For leaf rust testing, each isolate was developed from a single pustule, and purity was tested by inoculating a set of standard host differential lines as described by McCallum and Seto-Goh (2005). Three seedlings per cell were planted in a clump which was randomly spaced in a tray. Spores were suspended in light mineral oil at a concentration of ∼6 × 10^6^ spores/mL (Bayol, Esso Canada, Toronto, ON) and sprayed onto seedlings at the 1- to 2-leaf stage. Plants were allowed to dry for 1 h and then incubated in the dark at 20 ± 4°C with 100% relative humidity for 24 h. Plants were then transferred to a greenhouse and grown under a 16-h photoperiod at 20 ± 4°C until disease development. Infection types for both leaf rust and stem rust were recorded as described by Stakman et al. (1962) and McIntosh et al. (1995), with some modifications, at 14 days after inoculation. And the levels of infection types were: “0” = immune response, “;” = hypersensitive flecks, “1” = small uredinia with necrosis, “2” = small to medium sized uredinia with chlorosis or necrosis, “3” = medium sized uredinia without chlorosis or necrosis, and “4” = abundant large uredinia without chlorosis or necrosis. The symbols “-” and “+” indicate slight variations in the expression of an infection type. Scores of 0–2 were classified as resistant and scores of 3–4 as susceptible.

### Evaluation of Agronomic Performance

Lines RE21 and RE62 were evaluated for the following agronomic traits: plant height, spike length, number of spikelets per spike, days to heading (the period from transplanting to heading), and 1,000-kernel weight. Three plants of each line were grown in 6-inch pots in the greenhouse under controlled conditions of 15°C for 8 h in the dark followed by 20°C for 16 h of light. A complete randomized block with three replications was designed for the evaluation. Statistical analyses of the agronomic trait differences between the parents and two lines were conducted using ANOVA tests (Software package 21.0, SPSS).

## Results

### Production and Chromosome Composition of F_1_ Hybrids

**FIGURE 1:**
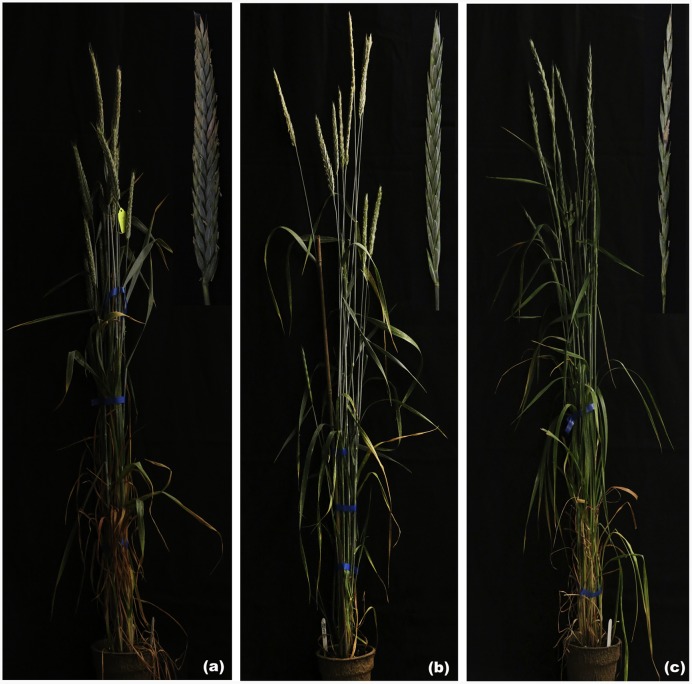
**The F_1_ hybrid and its parents. (a)** T182 and its spike. **(b)** F_1_ hybrid and its spike. **(c)** 8801 and its spike.

**FIGURE 2:**
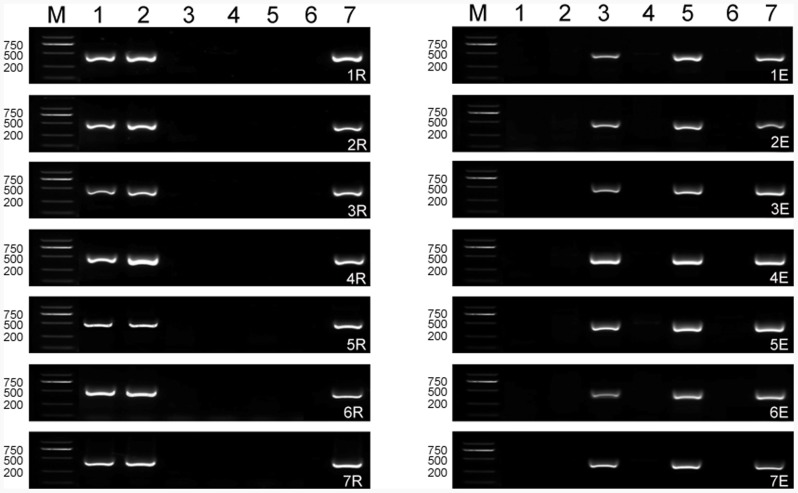
**Electrophoresis patterns of chromosome-specific molecular markers 1R to 7R and 1E to 7E in the F_1_ hybrid and its parents. M** Marker; **1** Imperial rye; **2** T182; **3** 8801; **4** Chinese Spring; **5** *Thinopyrum elongatum;* **6** Langdon; **7** F_1_ hybrid.

**FIGURE 3:**
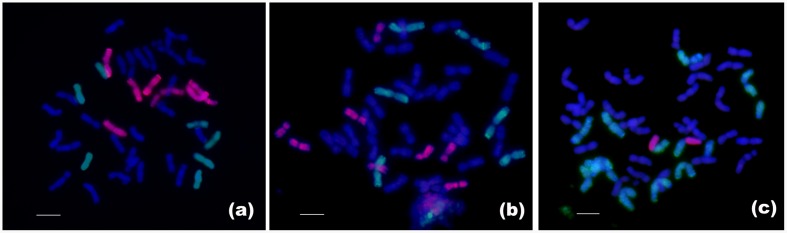
**Chromosome identification of the F_1_ hybrid and lines RE21 and RE62 by multicolor GISH. (a)** multicolor GISH pattern of F_1_ hybrid, consisting of 28 durum wheat chromosomes **(blue)**, 7 rye chromosomes (green), and 7 *Th. elongatum* chromosomes (red). **(b)** Line RE21 with 14 A- and 14 B-chromosomes of durum wheat plus 6 R-chromosomes of rye (green) and 8 E-chromosomes of *Th. elongatum* (red). **(c)** Line RE62 with 14 A- and 14 B-chromosomes of durum wheat, 12 R-chromosomes of rye (green), and 2 R-E translocated chromosomes. Bar = 10 μm.

Through extensive hybridization of the synthesized hexaploid 8801 with the triticale cultivar T182, F_1_ hybrids were obtained by embryo rescue. The phenotype of F_1_ hybrid was intermediate between the parental species but was sterile (**Figure 1**). PCR detected all seven E and R chromosomes in F_1_ hybrid (**Figure 2**). Multicolor GISH revealed the chromosome constitution in the F_1_ hybrid to be (*2n* = 6*x* = 42, AABBRE), composed of 28 wheat chromosomes (blue), 7 rye chromosomes (green), and 7 *Th. elongatum* chromosomes (red) (**Figure 3A**). GISH verified the successful production of a true F_1_ hybrid.

### Chromosome Constitution Analysis of Trigeneric Hybrid Lines RE21 and RE62 by Multicolor GISH

The genomic DNAs of *Th. elongatum* and Imperial rye were used as probes, and the genomic DNA of Chinese Spring was used as blocking DNA. In F_6_–F_8_ generations, multicolor GISH revealed that line RE21 had 42 chromosomes, consisting of 28 durum wheat chromosomes, 6 rye chromosomes, and 8 *Th. elongatum* chromosomes (**Figure 3B**). Line RE62 had 42 chromosomes consisting of 28 durum wheat chromosomes, 12 rye chromosomes, and 2 rye-*Th. elongatum* translocation chromosomes (**Figure 3C**).

### Chromosome Identification of Lines RE21 and RE62 Using Chromosome-Specific Markers

**FIGURE 4:**
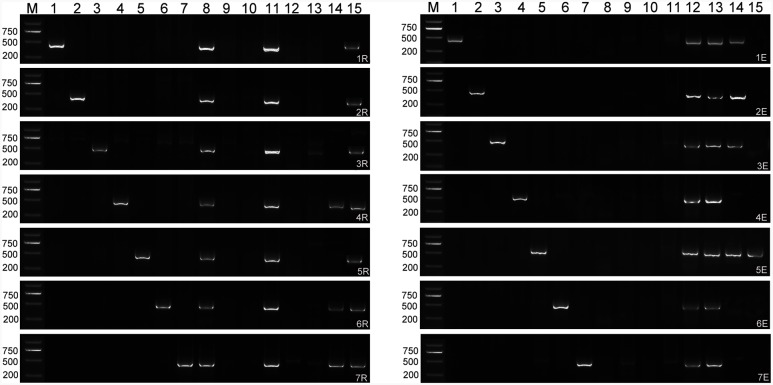
**PCR amplification of rye chromosome–specific markers from 1R to 7R and *Th. elongatum* chromosome–specific markers from 1E to 7E in the F_8_ generation of lines RE21 and RE62. M** marker; **1–7** Chinese Spring-Imperial addition lines (left), Chinese Spring-*Th. elongatum* addition lines (right); **8** Imperial; **9** Chinese Spring; **10** Langdon; **11** T182; **12** 8801; **13** *Th. elongatum*; **14** RE21; **15** RE62.

Individual chromosomes of lines RE21 and RE62 were confirmed by analysis with five rye chromosome markers and three *Th. elongatum* chromosome markers for each chromosome in F_6_–F_8_ generations. Analysis of line RE21 PCR products revealed specific bands for chromosomes 4R, 6R, and 7R and chromosomes 1E, 2E, 3E, and 5E, indicating that this line contained 14 A-chromosomes, 14 B-chromosomes, three pairs of R-chromosomes (4R, 6R, and 7R), and four pairs of E-chromosomes (1E, 2E, 3E, and 5E), for a total chromosome number of *2n* = 42 (**Figure 4**). PCR products from line RE62 showed bands for all seven rye chromosomes plus 5E-specific bands, indicating the presence of R/E chromosome translocations in this line (**Figure 4**). The PCR results of chromosome constitutions for line RE21 and RE62 were consistent with the GISH analysis.

### FHB Resistance

**FIGURE 5:**
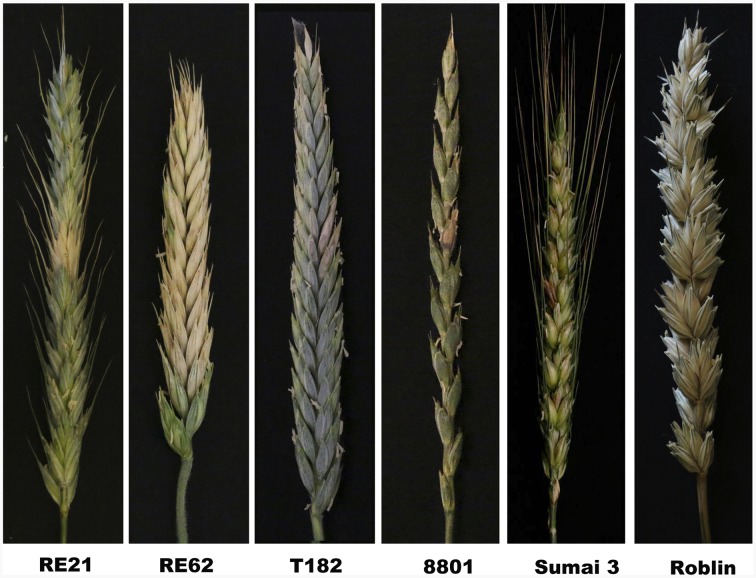
**Disease symptoms of experimental materials at 21 days after inoculation with *Fusarium graminearum***.

Following the establishment of the stable and fertile lines RE21 and RE62 in 2014 and 2015, the lines were screened for FHB resistance in the field and greenhouse. In resistant plants, the fungal infection was restricted to the central inoculated spikelet and did not spread up or down in the spike (**Figure 5**). The mean FHB infection rate was calculated based on individual observations of infection percentage in various spikes of the inoculated plants.

Point inoculation was applied to plants located in field plots in China in 2014. Following point inoculation, the average infection rate of florets in RE21 was 4.53% compared with a natural infection rate of 4.80%. This level of resistance approached levels observed for lines 8801 and Sumai 3 and was significantly better than levels observed for lines T182 and An 8455 (**Table 1**). Screening under greenhouse conditions in Ottawa, ON, Canada in 2015, the infection rates of Line RE21 were similar to those obtained in the field. The infection rate was 5.23% in the F_7_ generation and 5% in the F_8_ generation. The level of resistance observed was found to be significantly greater than that observed in Roblin (nearly 100%) and T182 but nearly the same as observed in 8801 (**Table 1** and **Figure 5**). These results provided clear evidence that the R or E chromosomes potentially carry FHB resistance genes. However, line RE62 was more susceptible to FHB, exhibiting nearly the same level of resistance as observed in Roblin and increased susceptibility compared with its parent T182. FHB resistance in line RE21 may have originated from *Th. elongatum* chromosomes. Analysis of variance (ANOVA) demonstrated that line RE21 exhibited significantly more FHB resistance than the control lines with significant differences detected in two consecutive years.

**Table 1:** Fusarium head blight (FHB) resistance of lines RE21 and RE62 in the field and greenhouse in 2014 and 2015.

Line or cultivar	2014 Yangzhou, China, field		2015 Ottawa, ON, Canada, greenhouse	
	Infection spikelets (%)		Infection spikelets (%)	
	Injection	Natural	Injection I	Injection II
RE21	4.53 ± 0.25^∗∗^	4.80 ± 0.42^∗∗^	5.23 ± 0.44^∗∗^	5.00 ± .028^∗∗^
RE62	-	-	98.88 ± 0.12	97.97 ± 0.22
T182	9.67 ± 0.28	8.70 ± 0.47	10.09 ± 0.13	10.34 ± 0.21
8801	5.10 ± 0.41	4.47 ± 0.37	5.10 ± 0.27	4.77 ± 0.32
Sumai 3	5.00 ± 0.17	4.57 ± 0.12	4.77 ± 0.21	4.90 ± 0.18
An 8455	66.53 ± 0.11	66.73 ± 0.18	-	-
Roblin	-	-	97.92 ± 0.14	98.75 ± 0.17

*^∗∗^Significantly different from both of the two susceptible controls, An 8455 and Roblin, at *p* < 0.01 (ANOVA)*.

### Leaf Rust and Stem Rust Responses

**FIGURE 6:**
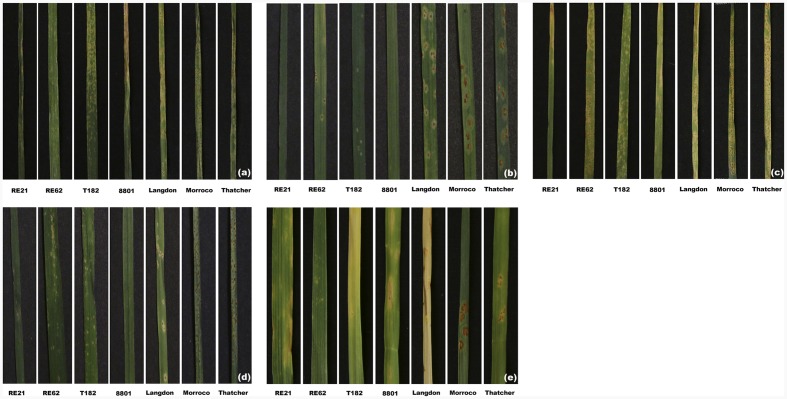
**Seedling leaf rust responses of RE21, RE62, and their parents against five Pt pathotypes. (a)** MBDS. **(b)** TJBJ. **(c)** MGBJ. **(d)** MBRJ. **(e)** TDBG.

Seedling rust responses were tested in Canada in 2015 and 2016 for lines RE21 and RE62 against one *Pgt* pathotype and five *Pt* pathotypes. Lines RE21 and RE62 exhibited infection types; 1- and; 0, respectively, against TTKSK (**Table 2**). The leaf rust response to the five Pt pathotypes in RE21 ranged from infection type 0 to 1+, and RE62 expressed infection types from 2- to 2+ (**Table 2**). Thatcher and Morocco were susceptible (infection type from 3+ to 4) to all five Pt pathotypes. **Figure 6** shows leaf rust responses to the five *Pt* pathotypes in RE21, RE62, the parents, and susceptible controls.

**Table 2:** Reactions of lines RE21, RE62, their parents and susceptible control cultivars to different races of *Puccinia triticinia* (*Pt*) and *P. graminis* f. sp. *tritici* (*Pgt*).

Line	*Pt* races/infection type^†^				*Pgt* races/infection type^†^	
	MBDS	TJBJ	MGBJ	MBRJ	TDBG	TTKSK
RE21	0+	1+	1	1+	0	;1-
RE62	2-	2-	2+	2-	2-	0;
T182	2+	2	2+	2-	0	0;
8801	0+	1	1+	1-	0	0;
Langdon	3+	3+	4	4-	3-	3+
Hoffman	nt	nt	nt	nt	nt	3
Thatcher	4	4	4	4	3+	nt
Morocco	4	3+	4	3+	4	nt

*^†^nt = not tested; 0 = immune response,; = hypersensitive flecks, 1 = small uredinia with necrosis, 2 = small to medium sized uredinia with chlorosis or necrosis, 3 = medium sized uredinia without chlorosis or necrosis, and 4 = abundant large uredinia without chlorosis or necrosis. The symbols “-” and “+” indicate slight variations in the expression of an infection type. Scores of 0–2 were classified as resistant and scores of 3–4 as susceptible*.

### Evaluation of Agronomic Traits

Results of agronomic trait evaluation under greenhouse conditions for lines RE21 and RE62 are shown in **Table 3**. Average plant height of line RE21 was significantly lower than that of both parents and line RE62. Spike lengths of both lines did not differ significantly from the parent line T182 but were significantly less than the other parent line 8801. Spikelet number per spike of both lines was significantly greater than that of either parent. The number of days to heading for both lines was significantly lower than the parents, and line RE21 headed earlier than line RE62. Both lines produced lower 1,000-kernel weight than T182 but higher than 8801, and the 1,000-kernel weight of line RE21 was lower than that of line RE62. Spike fertility was also lower in both lines than in either parent.

**Table 3:** Evaluation of agronomic traits of lines RE21, RE62, and their parents in the greenhouse.

Line	Plant height (cm)	Spike length (cm)	Spikelet number per spike	Days to heading	1,000-kernel weigh (g)	Spike fertility (%)
RE21	119.13 ± 1.73^c^	12.76 ± 0.58^c^	25.51 ± 0.58^c^	65.31 ± 1.15^c^	32.27 ± 0.45^c^	89.74 ± 1.95^c^
RE62	116.10 ± 1.16^ad^	12.67 ± 0.58^c^	29.33 ± 1.16^d^	69.33 ± 1.15^d^	56.13 ± 0.35^d^	89.23 ± 1.11^d^
T182	115.33 ± 1.53^a^	13.67 ± 0.57^a^	24.33 ± 1.53^a^	75.33 ± 1.15^a^	58.27 ± 0.21^a^	97.70 ± 0.43^a^
8801	165.33 ± 1.53^b^	17.33 ± 0.58^b^	23.33 ± 1.15^a^	93.01 ± 1.73^b^	26.47 ± 0.35^b^	95.14 ± 1.16^b^

*Different letters represent a significant difference at *p* < 0.01 (ANOVA)*.

## Discussion

The process of making crosses between different species or genera is commonly known as distant hybridization. Such hybrids have contributed to plant improvement, gene and genome mapping, and studies of chromosome behavior and evolution (Sharma, 1995). In recent years, distant hybridization between wheat and its wild relatives has attracted significant attention as a way to extend resources for genetic improvement in wheat breeding. A number of useful genes derived from wild relatives of wheat, such as leaf rust resistance gene (*Lr26*), stem rust resistance gene (*Sr31*), and stripe rust resistance gene (*Yr9*), had been transferred to common wheat (Villareal et al., 1991; McKendry et al., 1996). Following the development of distant hybridization, the technique of trigeneric hybridization has become a common method to transfer alien genes from wild species into cultivated wheat (Jiang et al., 1993). For example, the R genome derived from cultivated rye has served as an important resource for improving wheat and triticale because of its novel disease resistance and wide adaptation (Kang et al., 2012). Although it is difficult to synthesize the trigeneric hybrids via direct hybridization between common wheat and other two diploid wheatgrass, many trigeneric hybrids in *Triticeae* had been successfully obtained using different crossing combinations and embryo rescue (Kang et al., 2012), such as by crossing the intergeneric hybrids or amphiploid with other species (Cabrera and Martin, 1992; Li and Dong, 1993), by crossing barley (*Hordeum vulgare* L.) with triticale (Balyan and Fedak, 1989), or by crossing triticale with amphiploids involving wheat and other species (Fernandez-Escobar and Martin, 1989). The exploitation of crossing different amphiploid is an effective and rapid way to obtain trigeneric hybrids, which could be a useful bridge to transfer different alien characters into common wheat (Kang et al., 2012). This study, utilized embryo culture to obtain two *Triticum–Secale–Thinopyrum* trigeneric hybrid lines with different chromosome rearrangements between E and R chromosomes by crossing between triticale and the hexaploid amphiploid *T. trititrigia*. Both lines exhibited resistance to leaf rust and stem rust race Ug99, and line RE21 had additional resistance to FHB.

For successful distant hybridization, alien chromosome identification is essential for monitoring chromosome composition in hybrids. As techniques in molecular biology have evolved, the identification of alien chromosomes has greatly improved. GISH, using total genomic DNA of introgression species as a labeled probe, is the most efficient and widely used method to determine the origin of chromosomes or chromosomal fragments from wild species in the progenies of hybrids (Autrique et al., 1995). But this method has limitations, such as high cost and a long time involved, making it necessary to apply other methods to properly assess the nature and stability of newly added chromatin in progeny. Many studies have used PCR-based markers to detect individual chromosomes in a wheat background. For example, the specific simple sequence repeat markers (SSR), PCR-based landmark unique gene markers (so-called PLUG markers), and specific-locus amplified fragment (SLAF-seq) markers have been used to identify individual rye chromosomes or chromosomal segments in wheat background (Saal and Wricke, 1999; Li et al., 2015). The present study used GISH paired with SLAF-seq markers to identify rye and *Th. elongatum* chromosomes in the two hybrid lines and found the GISH results were consistent with the PCR analysis. Line RE21 had *2n* = 42 chromosomes, consisting of intact sets of A and B genomes from wheat plus 1E, 2E, 3E, 5E chromosomes from *Th. elongatum* and 4R, 6R, 7R chromosomes from rye; line RE62 also carried *2n* = 42 chromosomes, consisting of full sets of A and B genome, intact chromosomes 1R to 4R, 6R, and 7R, plus the translocation chromosomes 5R/5E.

*Thinopyrum elongatum*, a wild relative of wheat, has been suggested as a potentially novel source of resistance to FHB and may also harbor genes for FHB resistance (Fu et al., 2012). Some reports had indicated that the chromosome 7E from *Th. elongatum* contains resistance genes, such as the FHB resistance gene *Fhb7* and the rust resistance gene *Lr19* (Zhang et al., 2011; Guo et al., 2015). Jauhar et al. (2009) synthesized a stable durum disomic addition line by adding a single pair of 1E chromosomes originating from *Th. elongatum*, and observed resistance to FHB under greenhouse conditions. Kalih et al. (2014) reported that the dwarfing gene *Ddw1* detected on chromosome 5R of rye increases FHB severity. Line RE21 was highly resistant to FHB, possibly because chromosome 1E was present and chromosome 5R was absent. Line RE62 was more susceptible than either line RE21 or the parent T182 because the translocation occurred between chromosomes 5R and 5E. Notably, chromosome 5E may contain a major gene(s) that conveys susceptibility toward FHB (Fu et al., 2012). The presence of two chromosomes containing genes susceptible to FHB in line RE62 may explain the increase in susceptibility toward FHB. Additionally, the *Th. elongatum* chromosomes 1E and 7E, which contain FHB resistance genes, were not introgressed into line RE62.

It is well established that the short arm of rye chromosome 1R carries many disease resistance genes, including *Lr26*, *Sr31*, *Yr9*, and powdery mildew resistance genes (*Pm8*, *Pm17*) (Friebe et al., 1996; Geiger and Miedaner, 2009). Line RE62 exhibited significant resistance to leaf rust and stem rust race Ug99 owing to the presence of rye chromosome arm 1RS. In line RE21, even though chromosome 1R was replaced by *Th. elongatum* chromosome 1E, leaf rust resistance was still observed because *Th. elongatum* chromosome 3E carries leaf rust resistance gene *Lr24* (Schachermayr et al., 1995), and rye chromosomes 6R and 7R carry the resistance genes *Pr1* and *Pr2*, respectively (Wehling et al., 2003), thereby conferring resistance to leaf rust and stem rust race Ug99.

Under greenhouse conditions, several value-added characteristics including plant height, spike length, spikelets per spike, days to heading, and 1,000-kernel weight of lines RE21 and RE62 were measured. The average plant height of both lines RE21 and RE62 was greater than that of the parent T182, and plants of line RE21 were on average taller than plants of line RE62. The heading stage of both lines occurred earlier than either parent T182 and 8801, and the heading stage of line RE21 occurred earlier than line RE62. These results have confirmed that a dwarfing gene *Ddw1*, located on chromosome 5R, reduces plant height, increases FHB severity, and delays the heading stage (Kalih et al., 2014). Xiao et al. (2011) reported that the QTL for 1,000-kernel weight was located on rye chromosome arm 1RS. The 1,000-kernel weight observed in line RE21 was less than that of line RE62, possibly because chromosome 1R was replaced by 1E in RE21. The 1,000-kernel weight of line RE62 was similar to that of the parent line T182.

Compared with whole-chromosome transfer, development of translocation lines does incorporate small regions carrying useful genes into hexaploid wheat. Translocation lines are typically more genetically stable and their resistance is more durable compared with single-chromosome transfer lines (Zhao et al., 2013). The wheat-rye 1BL•1RS translocation line is particularly attractive to wheat breeders because several useful genes are located on 1RS (Singh et al., 1990). The development of translocation lines with small chromosome segments from wild species, especially those carrying several useful genes, is a promising direction for modern wheat breeding (Zhao et al., 2013). The application of distant hybridization toward the development of trigeneric hybrids may help establish evolutionary relationships among different genomes, offer the possibility to transfer different value-added trait into cultivated wheat, and produce translocations through the crossing or backcrossing of these plants to cultivated wheat (Li and Dong, 1993; Kang et al., 2012). Trigeneric hybrids seem to be less promising as potential crop, but they could be a useful bridge for the transference of chromatin into cultivated wheat. For example, Qi et al. (2016) produced a new wheat-rye-*Psathyrostachys* trigeneric hybrid carrying the wheat-rye 1BL•1RS translocation and a cryptic translocation involving a small chromosome segment with the stripe rust resistance gene from *P. huashanica*. In this study, we developed two *Triticum–Secale–Thinopyrum* hexaploid lines that carried resistance genes against FHB, leaf rust, and stem rust race Ug99. In the future, these two lines could be a useful bridge to develop the translocation lines carrying the wheat/rye recombinant chromosomes, wheat/*Th. elongatum* recombinant chromosomes or wheat/rye/*Th. elongatum* recombinant chromosomes and highly resistant to wheat diseases. Techniques such as radiation, molecular markers, and GISH will be useful for obtaining new lines in wheat breeding programs.

## Author Contributions

YD, GF, and JC participated in the study conception and design. HL, YD, and YaD contributed to DNA extraction and molecular marker identification. YD, DC, and WC performed hybridization. AX, YD, and HL analyzed the evaluation of disease resistance. YD, YaD, and YG analyzed the data. JC, YD, and GF wrote the manuscript. All authors approved the final version of the manuscript.

## Conflict of Interest Statement

The authors declare that the research was conducted in the absence of any commercial or financial relationships that could be construed as a potential conflict of interest.
