# Supplementary material for: Molecular Cytogenetic Characterization of two Triticum–Secale–Thinopyrum Trigeneric Hybrids Exhibiting Superior Resistance to Fusarium Head Blight, Leaf Rust, and Stem Rust Race Ug99
Source: Front Plant Sci. 2017 May 15;8:797. doi: 10.3389/fpls.2017.00797 (PMC5430057; doi:10.3389/fpls.2017.00797)
Supplement: Supplementary file 1 [file Table_1.DOCX]

**Table S1** The chromosome-specific molecular markers of rye and *Th. elongatum*.

| Specific Markers | Sequences of the Special Primers (5'–3') | | Amplified Chromosomes |
| --- | --- | --- | --- |
|  | Forward | Reverse |  |
| CMS1R-2 | TGCTGACGTGGGATGTTTTT | CTTTTGCTGATGGTTGGTAG | 1R |
| CMS1R-3 | TTATGAAGGCTTGAAGTGTG | ATCGTTTATGTAGTGTTGCG | 1R |
| CMS1R-8 | TACAACAACACAAATATTACCCCC | GGACTCGGAACAGGAGCAGA | 1R |
| CMS1R-9 | CACCACCACATTCATTCCAC | TCGGGGAGCCAGAAATTC | 1R |
| CMS1R-21 | TTGGTGTTCGTCTTGTTGA | TGTATTTTGCTTGGAACTTCT | 1R |
| CMS2R-6 | GAACTGGCAGTCTCAGATTG | AAACTCCAATCAGTATTATCGT | 2R |
| CMS2R-8 | CTTCAAGCAAAGCACAGAC | GGAGATAACACAAGGCAAC | 2R |
| CMS2R-13 | GGCTGCCCAATGAATGA | CATCACAATTGGCAAAGGG | 2R |
| CMS2R-14 | AGCACCTGTGTGACCGTAT | CAAGCGACTTCAAGGCATC | 2R |
| CMS2R-19 | TCCTTCTACATACTTGATT | ATAGTTACATTCCTTTCTT | 2R |
| CMS3R-4 | CCAGCTATCTTCCCCCACG | TACATATGGCACGAGAGGACC | 3R |
| CMS3R-5 | GTAGCTTCTGTTCCCGTGG | TTGTAAGCCAACTATTTTTCAG | 3R |
| CMS3R-6 | AGTGAGAGGCGACGGAAGG | CCCAATTTAACGCAAAACCTC | 3R |
| CMS3R-15 | ATACTTCGGTGCTCCCAAT | CACTCTCCCCTCTCATTCC | 3R |
| CMS3R-16 | TACGAGGGTTTACCTGTCGG | ATTCCTCTTATGAAGAGTCAACTAC | 3R |
| CMS4R-3 | GTTTCTACTAGGGGCGATTC | ATCTACACCAAAACACACGG | 4R |
| CMS4R-5 | CTCGGTGATGATGTTCGTCC | CTGCTTGCCTATAGCCCATG | 4R |
| CMS4R-6 | ATGGACATAATTGAAAAGGAAA | ACAAGGGAATGGTAAAAAAGTT | 4R |
| CMS4R-10 | GAATGTGTTTGGCCCTTTCGG | CAGTGCAAATCCCAATTCAGGG | 4R |
| CMS4R-22 | ACTCACTGCAGCATCCTCTAC | AGTATGTGGAGCCCAGTCTA | 4R |
| CMS5R-4 | TCATCCGAGTTACCGATTATT | CAGCACTTTCTGGGCTTCTT | 5R |
| CMS5R-5 | GTCTGCTCCAGGACCCTTG | TCGTCATACCCAAGAAAGGC | 5R |
| CMS5R-14 | GTCCACTTGTGGTTATTTCTCA | GGAGGGTCTTCCGCTTCTAT | 5R |
| CMS5R-27 | TCCGCTGACTATGTGTGTTAC | CTTAACTCGTTACTTGTCCTCA | 5R |
| CMS5R-28 | GGAAGGATTTGGAGCAAGGAGT | CGACGAGCAACAACAGAATGG | 5R |
| CMS6R-7 | GGTAGACTGCAACTCTTGGTGT | AGTTATTTGGTAATCACGAAGC | 6R |
| CMS6R-8 | AAGGAATCTATTCTATCCAGGC | GAGTTGTCAGCAGCTGGTAGT | 6R |
| CMS6R-10 | AGTTACTGAGGGAAGCTGGAAT | AAATAGCGGTCACCTAATGGGA | 6R |
| CMS6R-14 | TCGACTCATGAGAAGACCCA | TTCCTAGGATTTACCATTGACTT | 6R |
| CMS6R-16 | GCTTCTTCCTCGGTATCTCGTA | AATCTCCATCTATGCCGCAGTT | 6R |
| CMS7R-3 | TCTGCAATTCCAAATAGCGCA | TCTGGTTGTAACCTTCCCTGG | 7R |
| CMS7R-4 | GCCGCAATAGTGCAGTTCTC | GCAGTGCAGTTGTGTTCGTC | 7R |
| CMS7R-15 | GTTCATCAGATACAGAAAATGGTCA | TCGATCGACCGCTAACTGC | 7R |
| CMS7R-16 | TAGGCACTTCAACATCGGGG | TGGGCAACACAAAGGACACA | 7R |
| CMS7R-21 | GGCGTTGCACCTTATATGCC | GGGGGCAAGTTACTCGTCTTT | 7R |
| M1E_No.1 | TATCAAATAATATGTGCATG | CATGACAGAGATATGAAAAG | 1E |
| M1E_No.21 | CATCATATAAGAATGCAGGA | TCAACAGCACGCCAAC | 1E |
| M1E_No.30 | AAGTTCAAGTTTGCAGGTAC | AGCATTAGTATTTGAGAAGC | 1E |
| SLAF 2E-2 | GTGGCTCTGGTCTTGGGC | TTCATCTTTGTAAGTTTGTTGTCG | 2E |
| SLAF 2E-11 | TGGAGGAACTTGGTGGTGC | CGAATCCTACCGCCTGTGA | 2E |
| SLAF 2E-22 | TTTTGATGGCGACTTTAGG | CTTGGTTCCTGACCCTTT | 2E |
| SLAF 3E-1 | TGCGCTCGGAGATTTTAGTC | CCGTTCGGTGCAACATACAT | 3E |
| SLAF 3E-6 | GTCTACTTTAGCACCTCACTCA | AAATTCGTCCGGTTGTT | 3E |
| SLAF 3E-7 | CTACTTTAGCACCTCACTCAT | AAATTCGTCCGGTTGTT | 3E |
| SLAF 4E-3 | CAAAAGCACCTAACTCACCG | ACCAGGCTCTGTCCACTTAT | 4E |
| SLAF 4E-13 | GACAAGAAGACTGAAGATCGACA | TGACAGATTAGGACGAACGC | 4E |
| N28–4E603 | ATTCTCGCTCGCCACTAC | TGTATGCC ATCGAGCTATGTATT | 4E |
| SLAF 5E-3 | TCTCCCTCGTCTTCGTTTG | GGATATGAGCCCCAAGTTAG | 5E |
| SLAF 5E-6 | TGATTCGTAGGAGGAAGCAAG | TTGTTGAGTGCTACTTCCGTC | 5E |
| SLAF 5E-11 | GTGGCGACAGCACCTTCTT | CCAAAGGCAGGAGTAGGGTT | 5E |
| SLAF 6E-2 | GGGTCAGCCAACAACACAAG | ATCCACCTTTTTGCGTTCTG | 6E |
| SLAF 6E-9 | TGGAAGTCTATGCGGTCAAT | GTCGAGAACCCAAGTGGAAT | 6E |
| SLAF 6E-15 | AGTAATTGGAGACCCAGGCG | CAAATCAAACGACCATCACA | 6E |
| M7E_No.2 | AGCAAATAAAACGACAGT | GGTTTTCACCAATTACAG | 7E |
| M7E_No.4 | TTTTTCTGGTACTTACTAAC | TGTTCTGTGAGATGAATGTT | 7E |
| SLAF 7E-18 | TATCTTGGGCTCCCTACTCC | CTGGTTGAACAGCCGAAGGT | 7E |
